# Isolation and Identification of Putative Protein Substrates of the AAA+ Molecular Chaperone ClpB from the Pathogenic Spirochaete *Leptospira interrogans*

**DOI:** 10.3390/ijms19041234

**Published:** 2018-04-18

**Authors:** Joanna Krajewska, Zbigniew Arent, Michal Zolkiewski, Sabina Kędzierska-Mieszkowska

**Affiliations:** 1 Department of General and Medical Biochemistry, Faculty of Biology, University of Gdańsk, 80-308 Gdańsk, Poland; joanna.krajewska@biotech.ug.edu.pl; 2 University Centre of Veterinary Medicine JU-UAK, University of Agriculture in Kraków, 30-059 Kraków, Poland; zarent@ar.krakow.pl; 3 Department of Biochemistry and Molecular Biophysics, Kansas State University, Manhattan, KS 66506, USA; michalz@k-state.edu

**Keywords:** bacterial pathogens, ClpB, *Leptospira interrogans*, leptospirosis, molecular chaperones

## Abstract

Bacterial ClpB is an ATP-dependent Hsp100 chaperone that reactivates aggregated proteins in cooperation with the DnaK chaperone system and promotes survival of bacteria under stress conditions. A large number of publications also indicate that ClpB supports the virulence of bacteria, including a pathogenic spirochaete *Leptospira interrogans* responsible for leptospirosis in both animals and humans. However, the exact role of ClpB in bacterial pathogenicity remains poorly characterized. It can be assumed that ClpB, due to its role as the molecular chaperone, mediates refolding of essential bacterial proteins, including the known virulence factors, which may become prone to aggregation under infection-induced stresses. In this study, we identified putative substrates of ClpB from *L. interrogans* (ClpB_Li_). For this purpose, we used a proteomic approach combining the ClpB-Trap affinity pull-down assays, Liquid chromatography-tandem mass spectrometry (LC-MS-MS/MS), and bioinformatics analyses. Most of the identified proteins were enzymes predominantly associated with major metabolic pathways like the tricarboxylic acid (TCA) cycle, glycolysis–gluconeogenesis and amino acid and fatty acid metabolism. Based on our proteomic study, we suggest that ClpB can support the virulence of *L.* *interrogans* by protecting the conformational integrity and catalytic activity of multiple metabolic enzymes, thus maintaining energy homeostasis in pathogen cells.

## 1. Introduction

**Figure 1:**
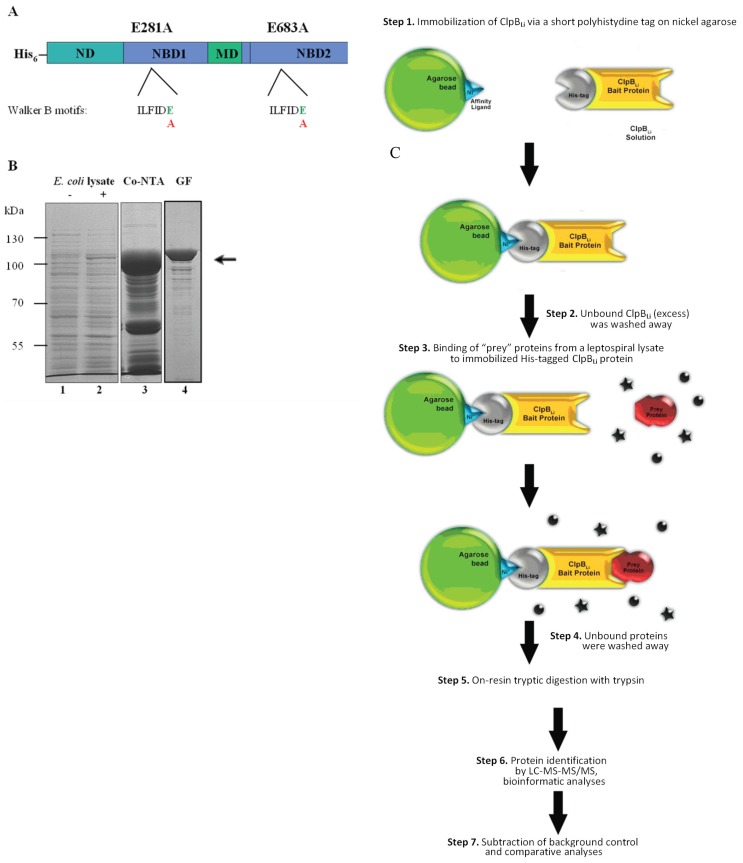
Schematic representation of the ClpB_Li_-Trap protein and the experimental strategy used in this study. (**A**) Domain structure of His_6_-tagged ClpB_Li_-Trap used for affinity pull-down experiments: N-terminal domain (ND) involved in recognition and binding of protein substrates, nucleotide binding domain 1 (NBD1), middle coiled-coil domain (MD) and nucleotide binding domain 2 (NBD2). The conserved sequences of the Walker B motifs are shown. The positions of residues within the Walker B motifs changed in this study are indicated. (**B**) The Coomassie blue-stained SDS-PAGE gel showing the lysates from *E. coli* cells carrying the recombinant plasmid expressing ClpB_Li_*-*Trap without induction (−) (lane 1) and induced with IPTG (+) (lane 2), and the representative fractions obtained following the cobalt affinity column (Co-NTA, lane 3) and gel filtration (GF, lane 4). The arrow indicates the position of His_6_-tagged ClpB_Li_-Trap (~98.5 kDa). The positions of protein markers (in kDa), PageRuler Prestained Protein Ladder (Thermo Scientific, Rockford, IL, USA), are shown on the left. (**C**) Overview of the experimental strategy used for trapping the putative protein substrates of ClpB_Li_. In the ClpB-Trap affinity pull-down experiments, Ni-NTA agarose was used instead of Co-NTA resin. The ● and * symbols indicate unbound proteins.

Bacterial ClpB is a molecular chaperone belonging to the Hsp100 subfamily of the AAA+ ATPases that cooperates with the DnaK chaperone system in solubilization and reactivation of aggregated proteins [1,2,3,4,5,6,7]. Like other Hsp100s, ClpB assembles into barrel-shaped hexamers in the presence of ATP [8]. Each ClpB monomer contains an N-terminal domain (ND) and two ATP-binding domains (NBD1, NBD2) with all characteristic and conserved sequence motifs of AAA+ ATPases, including Walker A and Walker B, and a coiled-coil middle domain (MD) inserted at the end of NBD1 (Figure 1A). ND of ClpB binds and recognizes protein substrates [9], whereas MD determines functional interactions with the DnaK chaperone system required for protein disaggregation both in vivo and in vitro [10,11]. It has been demonstrated that the mechanism of protein disaggregation mediated by ClpB involves the translocation of substrates through the central channel of the hexameric ring driven by the hydrolysis of ATP [5]. However, a recent study found that protein disaggregation might occur through one or two translocation steps, followed by rapid dissociation and rebinding of ClpB to a protein aggregate [12]. 

ClpB plays a crucial role not only in the survival of bacteria under stressful conditions [7,13], but also in supporting the virulence of some bacterial pathogens, including a pathogenic spirochete *Leptospira interrogans* [14,15,16,17,18] responsible for leptospirosis affecting animals and humans worldwide. It is estimated that over 1 million human cases of severe leptospirosis occur worldwide each year, with approximately 60,000 deaths from this disease [19,20]. It is worth noting that leptospirosis is also a serious economic problem in many countries, including the European Union. Each year, there are significant economic losses due to reproductive disorders in cattle, sheep, pigs, and horses that are linked to leptospirosis. Moreover, many serological and microbiological studies indicate a high rate of infections in domestic animals [21,22,23,24]. Despite the severity of leptospirosis and its global importance, the molecular mechanisms of the disease pathogenesis are not well understood, mainly due to a lack of standard genetic tools for use in *Leptospira* species. Identification of the *Leptospira* virulence factors and characterization of their activity is particularly important for understanding the mechanisms of the disease. To date, several virulence factors have been described in *Leptospira*, including the ompA-like surface lipoprotein, Loa22 [25], proteins involved in spirochete motility: FliY [26], FlaA2 [27] and LB139 [28], a heme oxygenase, HemO, which is essential for heme-iron utilization [29]; a catalase, KatE, required for resistance to extracellular oxidative stress [30]; phospholipase C, associated with *Leptospira*-induced macrophage death [31] and HtpG, the highly conserved molecular chaperone from the Hsp90 family [32]. A key virulence factor of *Leptospira* (common to all Gram-negative bacteria) is lipopolysaccharide (LPS), an important component of the bacterial outer membrane [33]. The molecular chaperone ClpB is also among the known leptospiral virulence factors because the *L. interrogans* ClpB mutant is avirulent, as opposed to its parental strain [18]. The deficiency of ClpB in *L. interrogans* also resulted in bacterial growth defects under oxidative and heat stress. As shown previously, the presence of ClpB in kidney tissues of *Leptospira-*infected hamsters and its immunogenicity [34] also support ClpB’s role in the pathogenicity of *Leptospira.* However, further studies are needed to elucidate ClpB’s role in virulence. In previous studies, we have demonstrated that the recombinant ClpB from *L. interrogans* (ClpB_Li_) displays the aggregate-reactivation activity that may support the survival of *L. interrogans* under host-induced stress, which is likely to cause denaturation and aggregation of pathogen proteins [35]. Interestingly, we found that ClpB_Li_ may mediate disaggregation of some aggregated proteins without the assistance of the DnaK system [36]. In this study, we constructed a His_6_-tagged ClpB_Li_-Trap variant with mutations of the Walker B motif in both ATP-binding domains to identify the putative substrates for ClpB_Li_ by using the protein–protein-interaction-based pull-down strategy [37] coupled with mass spectrometry (MS) analysis. The majority of ClpB-interacting proteins were associated with fundamental metabolic pathways like the TCA cycle, glycolysis–gluconeogenesis, or amino acid and fatty acid metabolism. Thus, our results suggest a possible role of ClpB_Li_ in controlling the energy metabolism of the *Leptospira* cell under stress. The remaining ClpB-interacting proteins were associated with other essential cellular processes like transcription, protein synthesis, cell wall and membrane biogenesis, spirochete motility, and chemotaxis. 

## 2. Results and Discussion 

**Figure 2:**
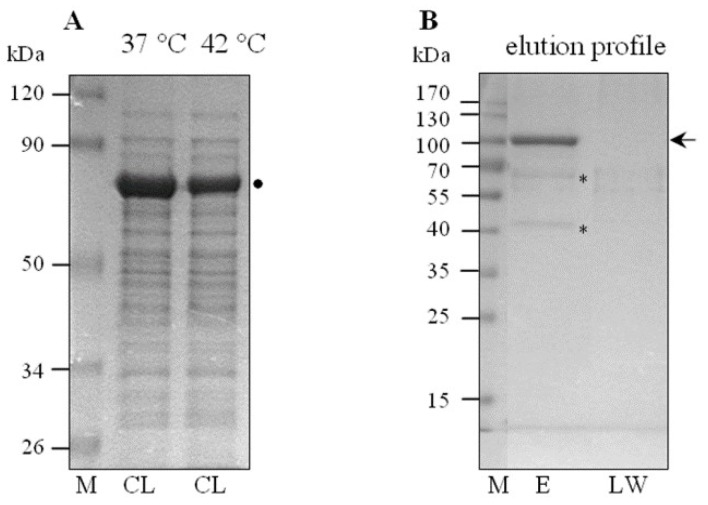
SDS-PAGE analysis of the lysates of *Leptospira* cells (CL) cultured at 37 °C or 42 °C (**A**) and a representative sample of the elution profile of the ClpB_Li_-Trap binding proteins (**B**); (M), Prestained Protein Molecular Weight Markers (kDa); Thermo Scientific. (E), the eluted fraction; (LW), the last wash fraction. 12.5% polyacrylamide gels separated using different run-time, were stained with Coomassie Brilliant Blue. The arrow indicates the position of the His_6_-tagged ClpB_Li_-Trap protein (~98.5 kDa). The * symbols indicate the putative ClpB_Li_ protein substrates, and the ● symbol indicates GroEL/DnaK identified by LC-MS-MS/MS analysis.

To reveal the underlying mechanism by which the ClpB chaperone may influence virulence traits in *L. interrogans*, we have attempted for the first time to identify the *Leptospira* proteins that can be recognized and potentially reactivated by ClpB_Li_ in cells under environmental stress, including changes in temperature. First, we produced a “substrate trap” variant of ClpB_Li_ (ClpB-Trap; Figure 1A) with mutations within the Walker B motif of both ATP-binding domains (E281A/E683A) based on the work of Weibezahn et al. [38]. These authors showed that ClpB from *E. coli* with the same mutations in the Walker B motifs binds ATP, but is deficient in the ATP hydrolysis and therefore forms stable complexes with its protein substrates. Additionally, ClpB-Trap was engineered to contain an N-terminal polyhistidine tag. After two-step purification (Figure 1B), His_6_-tagged ClpB_Li_-Trap was immobilized on nickel agarose beads and used to capture its potential substrates from the cellular lysates of *Leptospira* followed by mass-spectrometry-based proteomics. Our strategy for isolation and screening of the ClpB_Li_ substrates is summarized in Figure 1C. The lysates were prepared from *L. interrogans* serovar Copenhageni cultures exposed to thermal stress at two temperatures, 37 °C (mild heat shock conditions) and 42 °C (severe heat stress) (see Section 3.1). Equal amounts of the total protein lysates (1 µg) were analyzed by SDS-PAGE with Coomassie blue staining (Figure 2A). No apparent differences between the protein profiles obtained under the two heat shock conditions were observed. In the most prominent band of the gel, at approximately 70–80 kDa, we identified mostly GroEL by using LC-MS-MS/MS analysis, although DnaK was also present. Control samples were prepared in parallel with the primary samples (see Section 3.4) to test the effect of ClpB_Li_ binding to the agarose beads and its possible interactions with the endogenous proteins of *Leptospira*. In the case of the control samples (Figure 2B,E), the trapped proteins were eluted with 250 mM imidazole buffer and analyzed by SDS-PAGE and Coomassie blue staining. The last wash fractions (Figure 2B; LW) were also analyzed in the same way to confirm that all unbound proteins had been washed away from the agarose beads. 

The above experiments were also carried out using His_6_-tagged HtrA (~51-kDa) as bait (Table 1). The proteins bound to HtrA did not overlap with those captured by ClpB_Li_, with the exception of succinate dehydrogenase (Table 2). We believe that a comparison with HtrA, which is not related to ClpB and acts as an oligomeric periplasmic serine protease, further validates our strategy for identifying ClpB-specific interactions. 

**Figure 3:**
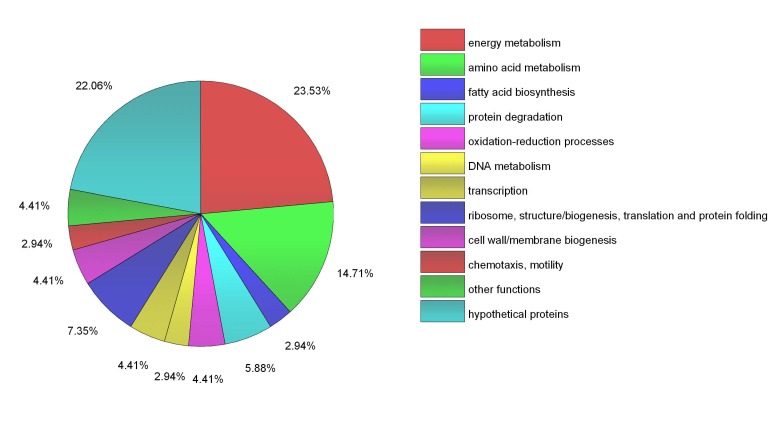
Functional classification of 68 identified proteins. The pie-chart created using OriginLab software (OriginPro 2016, Northampton, MA, USA) shows the distribution of these proteins into their biological processes in percentage.

Table 2 shows a list of all the proteins identified in this study as candidate substrates or partners of ClpB_Li_ after elimination of the background control sample (Table S1, Supplementary Materials) and the pie chart (Figure 3) shows the distribution of these proteins among different functional classes. In total, 68 proteins were identified as ClpB interactors, of which 62 proteins were associated with the lysate prepared from cells submitted to mild heat shock at 37 °C and six additional proteins were obtained when cells were heat-shocked at 42 °C (26 proteins were found in both these fractions). Among the potential ClpB substrates, 15 proteins were annotated as “hypothetical proteins.” The majority of the remaining identified proteins were involved in the central metabolism and energy production. Among them, 10 proteins were assigned to amino acid metabolism and six proteins (i.e., aconitate hydratase, citrate (Si)-synthase, malate dehydrogenase, succinate dehydrogenase flavoprotein subunit, citrate lyase, succinyl-CoA ligase) were involved in the TCA cycle. Another class of the ClpB-interacting proteins was enzymes involved in lipid metabolism (glycerol-3-phosphate dehydrogenase, 2,4-dienoyl-CoA reductase, acyl-CoA hydrolase, acyl-CoA dehydrogenases, acetyl CoA C-acetyltransferase, and biotin carboxylase). Several of the proteins identified in this study were linked to other essential cellular processes, such as ribosome biogenesis (30S ribosomal proteins: S3, S4 and S15), translation (elongation factor 4/LepA), redox homeostasis, or proteolysis. Interestingly, it has been shown that ClpB co-sediments with ribosomes isolated from *E. coli* cells exposed to heat shock at 45 °C and interacts with some ribosomal proteins [40].

Among the proteins identified in this study (see Table 2) was SAM-dependent methyltransferase, which catalyzes the methylation of biomolecules, including amino acids, proteins, and DNA. In addition, two identified proteins were directly associated with chemotaxis and spirochete motility, which support the *L. interrogans* virulence in the hamster model for leptospirosis [41]. The remaining proteins were associated with the cell wall or membrane biogenesis.

The identified proteins, including the enzymes of major metabolic pathways like the TCA cycle, glycolysis–gluconeogenesis, amino acid and lipid metabolism (Table 2), may require the assistance of ClpB_Li_ during heat shock. In fact, it has been demonstrated that key metabolic enzymes are heat-sensitive and aggregation-prone and therefore are often inactivated by stress [42]. Stress conditions induce structural destabilization, unfolding and, ultimately, aggregation of enzymatic components of the major metabolic pathways.

Interestingly, Fischer and co-workers [43] have recently found that the mitochondrial ClpXP protease in *Podospora anserin*a is mainly associated with enzymes involved in TCA cycle, amino acid and fatty acid metabolism, and subunits of electron transport chain complex. In the ClpXP complex, the ATPase ClpX is responsible for substrate recognition and contains structural domains homologous to those found in ClpB. Many proteins involved in energy metabolism and also in protein translation, transcription, DNA metabolism and fatty acid metabolism, were also reported as substrates of the *Staphylococcus aureus* ClpC chaperone that is an ATP-dependent Hsp100 chaperone like ClpB [44]. 

Furthermore, we have previously found that ClpB_Li_ is able to reactivate thermally inactivated fructose-bisphosphate aldolase, one of the identified metabolic enzymes, even in the absence of the DnaK chaperone system from *E. coli* [36]. 

We propose that the key metabolic enzymes are the main substrates for the molecular chaperone ClpB_Li_ and preservation of their activity under stress conditions depends on the ClpB_Li_ disaggregase activity. It is likely that the metabolic enzymes have an important impact on the growth of *Leptospira* cells and the leptospiral pathogenicity. We suggest that ClpB_Li_ influences virulence traits in *L. interrogans* mainly through preservation of the activity of metabolic enzymes.

The remaining identified ClpB_Li_ substrates, 2-dehydro-3-deoxyphosphooctonate aldolase responsible for biosynthesis of the oligosaccharide core of LPS, an essential virulence factor in all Gram-negative bacteria, the chemotaxis proteins, or a membrane lipoprotein LruA (LipL71), may support the *Leptospira* virulence. Other potential substrates of ClpB_Li_ include proteins involved in the mRNA metabolism (polyribonucleotide nucleotidyltransferase) or transcription (DNA-directed RNA polymerase α subunit, ArsR family transcriptional regulator). Thus, ClpB_Li_ could indirectly influence gene expression in leptospiral cells. It is noteworthy that Arifuzzaman et al., 2006 [45] observed interactions between a multi-subunit complex of RNA polymerase (RNAP) from *E. coli* and some chaperones, including ClpB. Those data suggest that not only DnaK, but also ClpB, may assist the assembly of RNAP.

In summary, we performed the first identification of the potential protein substrates of ClpB_Li_. The majority of these proteins is associated with energy-generating metabolism and may have an important impact on the grown and pathogenicity of *Leptospira*. Further in vitro studies will determine whether the proteins identified in this work interact directly with ClpB or if they share interacting partners with the chaperone. Our results suggest a possible role of ClpB_Li_ in maintaining the energy-generating metabolism of the *Leptospira* cell and strongly support the ClpB’s importance in the leptospiral virulence. We believe that our results help explain the previously established role of the molecular chaperone ClpB in supporting bacterial pathogenicity.

## 3. Materials and Methods

### 3.1. Leptospira Strain, Growth Conditions, and Cell Lysate Preparation

*L. interrogans* serovar Copenhageni strain B42 was grown in liquid Ellinghausen McCollough Jonhson and Harris medium (EMJH) at 30 °C until mid-exponential phase (OD_420_ = ~0.3) then transferred to 37 or 42 °C for 4 or 2 h, respectively (protein aggregate formation). After exposure to thermal stress, a total of 100 mL of cells were harvested by centrifugation at 6000× *g* for 10 min at room temperature and cell lysates were prepared as previously described [46] with some modifications. Briefly, leptospires were washed twice with phosphate-buffered saline (PBS, pH 7.4), 5 mM MgCl_2_ and resuspended in lysis buffer (10 mM Tris/HCl, pH 8.0, 2 mM EDTA, 25 mM NaCl, 1 mM PMSF protease inhibitor) containing 1mg/mL of lysozyme. The suspension was incubated for 5 min at 4 °C and then subjected to three cycles of freezing (−80 °C) and thawing (room temperature) with vigorous vortexing. Next, DNase I (to a final concentration of 5 µg/mL) was added and the cell suspensions were incubated on ice for 20 min, sonicated with 20% amplitude in 5 s pulses for 30 s using a microtip Vibra Cell sonicator. The insoluble materials (unbroken cells and cell debris) were removed by centrifugation at 6000× *g* for 10 min at 4 °C. The soluble supernatants (total cell lysates), including unfolded or aggregated proteins, were used for screening protein substrates/partners of ClpB_Li_. Protein concentration in each cell lysates was determined by the Bradford method [47] using BSA as a standard. For assessment of the lysis efficiency, SDS-PAGE electrophoresis (Bio-Rad, Hercules, CA, USA) was performed as described previously [48] using 12.5% polyacrylamide gels followed by staining with Coomassie Brillant Blue. 

### 3.2. Construction of ClpB_Li_-Trap Mutant

To construct a ClpB-Trap variant useful for trapping ClpB substrates, we replaced the conserved glutamic acid in each of the two Walker B motifs (E281 and E683 in the NBD1 and NBD2, respectively) with alanine. The mutations were introduced by the QuickChange II site-directed mutagenesis method (Agilent Technologies, Santa Clara, CA, USA) using primers with the desired mutation and Pfu Turbo DNA polymerase (Agilent Technologies). The pET28ClpB_Li_ construct [34] was used as the template DNA in a mutagenic PCR reaction. The generated construct was confirmed by DNA sequencing (Genomed S.A., Warsaw, Poland).

### 3.3. Purification of ClpB_Li_-Trap (E281A/E683A)

The His_6_-tagged ClpB-Trap protein was overproduced from the recombinant plasmid pET28b in *E. coli* BL21 (DE3) strain (Novagen/Merck, Darmstadt, Germany) and purified in two steps using HisPur cobalt resin (Thermo Scientific, Rockford, IL, USA) followed by Superdex 200 gel filtration as previously described [34]. Fractions containing ClpB_Li_ were identified by SDS-PAGE and staining with Coomassie blue. ClpB concentration was estimated from absorption at 280 nm using for ClpB_Li_ the extinction coefficient ε^0.1%^ = 0.445 (mg/mL)^−1^ cm^−1^ calculated from its amino acid composition by ProtParam [49].

### 3.4. Affinity Pull-Down Assay and MS Analysis 

His_6_-tagged ClpB_Li_-Trap (1.5 μM) in buffer A (50 mM Tris-HCl, pH 8.0, 300 mM NaCl, 20 mM imidazole) was incubated with 15 μL of Ni-NTA agarose (Macherey-Nagel, Düren, Germany) suspended in the same buffer A for 3 h at 4 °C. Agarose beads were washed twice with buffer A and then the soluble protein fractions (~100 µg proteins) prepared from *L*. *interrogans* cultures submitted to thermal stress as described above were added. We used an excess of His-tagged ClpB_Li_-Trap over potential binding proteins (prey proteins). Thus, competition for binding to Ni-NTA between the prey proteins and ClpB is unlikely. After a 30-min incubation in the presence of 2 mM ATP at room temperature, the agarose beads were washed with buffer A containing 2 mM ATP (15 times with 200 μL), then eluted with buffer A containing 250 mM imidazole to test ClpB_Li_ binding efficiency to the beads or suspended in water and used as a “bead proteome” for identification of proteins interacting with ClpB_Li_. For this purpose, LC-MS-MS/MS analysis of tryptic peptides obtained after trypsin cleavage of the separated proteins was performed at the MS LAB IBB PAN (Warsaw, Poland). The resulting MS/MS spectra were submitted to the program Mascot and searched against the NCBI-nr database (57,412,064 sequences and 20,591,031,683 residues). The search was restricted to *L. interrogans* proteins (104,694 sequences). Positive hits were identified with at least two unique peptides present in two independent biological replicates of each sample with a Mascot ion score above 30. Proteins found in the background control sample (the agarose beads incubated with the total cell lysates, described above, in the absence of the His_6_-tagged ClpB_Li_-Trap protein; Table S1, Supplementary Materials) were eliminated from the set of candidate ClpB binding proteins. 

As another control, we performed an affinity pull-down assay using a protein unrelated to ClpB, His_6_-tagged HtrA (1.5 µM), as bait (Table 1), and the same experimental conditions as described above, with one exception—namely, no ATP was added to the buffers. 

The two control experiments described above were performed to ensure that the detected interactions between His_6_-tagged ClpB_Li_ and proteins from the leptospiral lysates were linked to the ATP-dependent function of ClpB and not to nonspecific binding.

## Figures and Tables

**Table 1:** Control protein profile eluted from Ni^2+^-NTA agarose using His_6_-tagged HtrA as bait.

Protein Name	Gene ID ^a^/Gene Name Accession Number	Molecular Mass (kDa) ^b^	Sequence Coverage (%)	Matched Peptides	Score ^c^
50S ribosomal protein L19	LIC11559/*rplS* gi|446995403	15.5	13	2	56
Succinate dehydrogenase flavoprotein subunit	LIC12002/*sdhA* gi|45657855	70.9	5	4	287
Hypothetical protein	gi|446175654	15.3	9	2	65
LipL45	LIC10123 gi|45600754	42.3	6	2	173
LipL46	LIC11885 gi|447001777	34.7	30	8	380
Conserved hypothetical protein	LIC11848 gi|45600951	32.1	9	2	102

^a^ Gene ID was based on Open reading frames (ORFs) of the genome sequence of *L. interrogans* serovar Copenhageni deposited in GenBank under accession numbers AE016823 (chromosome I) and AE016824 (chromosome II) [39]. ^b^ Theoretical molecular mass (kDa) was determined by Mascot. ^c^ Represent MS/MS ion scores determined by peptide mass fingerprinting. Only scores that were deemed to be significant by Mascot analysis (*p* < 0.05) are given.

**Table 2:** Proteins of *L. interrogans* serovar Copenhageni bound to His_6_-tagged ClpB_Li_-Trap.

Function and Protein Name	Gene ID ^a^/Gene Name Accession Number	Molecular Mass (kDa) ^b^	Sequence Coverage (%) 37/42 °C	Matched Peptides 37/42 °C	Score ^c^ 37/42 °C
*Energy metabolism (16)* *					
Fructose-bisphosphate aldolase	LIC12233 gi|45658082	37.8	14/-	3/-	180/-
Triosephosphate isomerase	LIC12094/*tpiA* gi|45601183	27.4	11/-	3/-	128/-
Alcohol dehydrogenase	LIC10253/*adh* gi|45599391	45.9	25/4	10/2	351/114
Putative citrate lyase	LIC11194 gi|45600315	38.4	9/-	3/-	146/-
Aconitate hydratase	LIC20249/*acnA* GI:45655824	82.3	12/5	9/4	375/212
Type II citrate synthase	LIC12925/*gltA* gi|45601997	48.6	-/4	-/2	-/118
Matate dehydrogenase	LIC11781/*mdh* gi|45600887	35.1	37/19	8/4	830/273
Succinate dehydrogenase flavoprotein subunit	LIC12002/*sdhA* gi|45657855	71.0	11/2	5/2	229/95
Acyl-CoA hydrolase, thioesterase family protein	LIC11758 gi|45600864	16.0	17/-	2/-	119/-
2,4-dienoyl-CoA reductase	LIC11729/*fadH* gi|45600834	73.8	9/-	4/-	283/-
Electron transfer flavoprotein subunit alpha	LIC10360/*etfA* gi|45656263	28.2	43/13	9/2	611/142
Acyl-CoA dehydrogenase	LIC10583/*acd* gi|45599716	48.7	5/-	3/-	185/-
Acyl-CoA dehydrogenase	LIC13009/*acd* gi|447196883	55.7	-/8	-/3	-/106
Acetyl CoA C-acetyltransferase	LIC12795/*phbA* gi|446701619	48.0	10/-	3/-	127/-
Succinyl-CoA ligase/synthetase subunit β	LIC12573/*sucC* gi|446613340	40.4	14/17	5/3	247/165
2-oxoglutarate dehydrogenase E1 component	LIC12474/*odhA*	103.6	5/7	7/9	275/360
*Amino acid metabolism (10)* *					
Acetolactate synthase small subunit	LIC11410/*ilvH* gi|45600525	18.0	15/-	2/-	65/-
*N*-acetyl-gamma-glutamyl-phosphate reductase	LIC11746/*argC* gi|45600852	37.8	5/-	2/-	55/-
S-adenosyl-l-homocysteine hydrolase	LIC20083/*ahcY* gi|45655666	48.7	4/5	2/2	88/73
Putative branched-chain amino acid aminotransferase	LIC13496/*ilvE* gi|45602553	35.1	17/-	4/-	207/-
Pyridoxal phosphate-dependent aspartate aminotransferase superfamily (ABHA synthase)	LIC12168/*aspC* gi|45601258	44.6	-/6	-/2	-/95
B12-dependent methionine synthase	LIC20085/*metH* gi|45655668	142.5	1/1	2/2	48/79
3-isopropylmalate dehydrogenase	LIC11768/*leuB* gi|45657634	39.0	19/-	6/-	355/-
d-3-phosphoglycerate dehydrogenase/4-phosphoerythronate dehydrogenase	LIC11992/*serA* gi|45601085	42.3	13/-	3/-	152/-
Cysteine desulfurase	LIC20204/*csdB* gi|45655784	43.8	15/-	3/-	130/-
Ketol-acid reductoisomerase	LIC13393/*ilvC* gi|45602456	35.4	16/13	5/4	271/189
Cysteine synthase	LIC12082/*cysK* gi|446567601	33.2	14/-	2/-	104/-
*Nucleotide biosynthesis (1)* *					
Inosine-5′-monophosphate dehydrogenase	LIC11919/*guaB* gi|45601019	56.0	6/-	2/-	67/-
Fatty acid biosynthesis (2) *					
FAD dependent oxidoreductase/glycerol-3-phosphate dehydrogenase	LIC11699/*glpD* gi|45600804	62.0	7/3	3/2	168/95
Biotin carboxylase	LIC11518/*accC* gi|446487065	102.4	-/2	-/2	-/68
*Inorganic ion transport, homeostasis (1)* *					
Potassium transporter TrkA	LIC13175/*trkA* gi|45602242	26.7	8/-	2/-	107/-
*Protein degradation (4)*					
Cysteine protease (papain family cysteine protease)	LIC20197 gi|45602748	87.8	6/5	3/2	192/118
Aminopeptidase N	LIC12591/*pepN* gi|45601672	102.2	6/-	3/-	68/-
PDZ domain protein, trypsin-like peptidase domain protein/Serine protease MucD precursor	LIC12812/*mucD* gi|45601887	41.2	23/12	5/2	257/176
ATP-dependent ClpP protease ATP-binding subunit ClpX	LIC11418/*clpX* gi|456986981	46.7	3/-	2/-	168/-
*Oxidation-reduction processes (3)*					
Molybdopterin oxidoreductase (4Fe-4S-cluster domain protein)	LIC10874 gi|45656765	113.6	21/-	14/-	713/-
GMC family oxidoreductase	LIC10037 gi|45655951	58.6	3/-	2/-	69/-
Rubrerythrin domain protein	LIC20205 gi|446945174	30.6	11/21	6/12	323/535
*DNA metabolism (2)* *					
DNA-binding ferritin-like protein	LIC10606/*dps* gi|45599739	18.2	47/23	5/3	397/228
Recombinase RecA	LIC11745/*recA* gi|446426865	39.8	6/3	5/6	240/371
*Transcription (4)* *					
ArsR family transcriptional regulator	LIC11617 gi|45600728	11.2	32/35	2/2	75/206
DNA-directed RNA polymerase subunit alpha	LIC12846/*rpoA* gi|45601920	36.7	36/8	13/2	474/165
Polyribonucleotide nucleotidyltransferase/polynucleotide phosphorylase	LIC12701/*pnpA* gi|45601779	76.6	21/4	14/2	748/130
Transcription termination factor Rho	LIC12636/*rho* gi|45601716	53.8	12/2	6/2	375/129
Ribosome structure/biogenesis, translation and protein folding (4)*					
30S ribosomal protein S15	LIC12702/*rpsO* gi|45601780	10.3	-/27	-/2	-/78
30S ribosomal protein S4	LIC12847/*rpsD* gi|446057405	24.1	7/-	4/-	133/-
30S ribosomal S3	LIC12867/*rpsC* gi|446452098	25.7	14/-	2/-	86/-
Elongation factor 4/LepA	LIC12010/*lepA* gi|45601104	67.3	11/-	6/-	339/-
*Regulatory function (1)* *					
SAM-dependent methyltransferase	LIC12190/*smtA* gi|45601280	26.1	17/-	2/-	109/-
*Cell wall/membrane biogenesis (3)* *					
2-dehydro-3-deoxyphosphooctonate aldolase/3-deoxy-8-phosphooctulonate synthase	LIC11541/*kdsA* gi|45600653	32.2	16/-	3/-	190/-
LipL71/LruA	LIC11003/*lipL71* gi|45600127	62.1	16/6	6/3	237/199
Rod shape-determining protein/cell shape determining protein, MreB/Mrl family	LIC11258/*mreB* gi|456985405	37.0	9/-	4/-	275/-
*Chemotaxis, motility (2)* *					
Chemotaxis protein	LIC12456/*cheA* gi|476492777	120.0	6/-	6/-	275/-
Methyl-accepting chemotaxis protein	LIC12921/*mcpA* gi|45601994	76.8	15/-	10/-	605/-
*Hypothetical proteins (15)*					
Conserved hypothetical protein (with the CBS domain)	LIC12236 gi|45601326	16.6	29/-	3/-	142/-
Conserved hypothetical protein (metallo-beta-lactamase superfamily hydrolase)	LIC12478 gi|4560156	35.4	10/-	2/-	76/-
Conserved hypothetical protein (with PIN domain)	LIC10215 gi|45656120	37.0	10/14	3/2	185/270
Hypothetical protein (TPR protein)	LIC10125 gi|45656035	135.3	4/3	4/3	186/212
Conserved hypothetical protein (helicase C-terminal domain protein)	LIC11405 gi|45600520	76.9	5/3	3/2	162/124
Conserved hypothetical protein	LIC11274 gi|45600394	43.2	5/-	2/-	94/-
Conserved hypothetical protein (region ClpX-like)	LIC10558 gi|45599691	17.3	15/-	2/-	115/-
Conserved hypothetical protein (carbohydrate-binding protein, F5/8 type C domain protein)	LIC20001 gi|45602557	91.0	9/-	5/-	205/-
Hypothetical protein	LIC13428 gi|45659245	54.9	15/10	6/5	438/284
Conserved hypothetical protein	LIC10017 gi|45599164	34.3	-/3	-/2	-/88
Conserved hypothetical protein (PaaI family thioesterase)	LIC11209 gi|446570840	15.1	-/23	-/2	-/127
Hypothetical protein (aminotransferase)	LIC12198 gi|45601288	41.6	9/-	2/-	144/-
Hypothetical protein	LIC10235 gi|45656140	10.9	22/-	2/-	126/204
Hypothetical protein (HEAT repeat domain-containing protein)	LIC10411 gi|446594877	17.2	14/-	2/-	140/-
Conserved hypothetical protein (ATPase)	LIC12581 gi|45601662	18.3	30/-	2/-	151/-

^a^ Gene ID was based on ORFs of the genome sequence of *L. interrogans* serovar Copenhageni deposited in GenBank under accession numbers AE016823 (chromosome I) and AE016824 (chromosome II) [39]. ^b^ Theoretical molecular mass (kDa) was determined by Mascot. ^c^ Represent MS/MS ion scores determined by peptide mass fingerprinting. Only scores that were deemed to be significant by Mascot analysis (*p* < 0.05) are given. (*), proteins (functional categories) indicated as the candidate ClpB_Li_ substrates.

## Supplementary Materials

Supplementary materials can be found at http://www.mdpi.com/1422-0067/19/4/1234/s1.
