# Supplementary material for: Isolation and Identification of Putative Protein Substrates of the AAA+ Molecular Chaperone ClpB from the Pathogenic Spirochaete Leptospira interrogans
Source: Int J Mol Sci. 2018 Apr 18;19(4):1234. doi: 10.3390/ijms19041234 (PMC5979558; doi:10.3390/ijms19041234)
Supplement: Supplementary file 1 [file ijms-19-01234-s001.pdf]

Supplementary Material

Table S1. Background proteins bound to Ni^{2+}-NTA agarose.

Protein name	Gene ID[a]/ gene name Accession number	Molecular mass (kDa)[b]	Sequence coverage (%)	Matched peptides	Score[c]
Catalase	LIC12032/*katE* gi|488106341	54.8	22	8	571
DnaK	LIC10524/*dnaK* gi|1628635	69.1	11	5	356
GroEL	LIC11335/*groEL* gi|45600451	58.0	15	8	372
Peptidyl-prolyl *cis-trans* isomerase	LIC11731/*slyD* gi|45600836	17.4	41	5	632
LipL32	LIC11352/*lipL32* gi|73300742	29.2	29	5	501
LipL41	LIC12966/*lipL41* gi|42628217	34.0	12	3	191
Flagellar filament core protein, FlaB	LIC11889/*flaB* gi|45657404	31.5	40	8	827
Flagellar filament outer layer protein, FlaA-1	LIC10788 gi|45656681/ *flaA-1*	35.0	10	3	207
Flagellin protein	LIC11890 gi|45657753	31.3	22	8	1000
Elongation factor Tu	LIC12875/*tuf* gi|446963314	43.7	18	5	317
DNA-directed RNA polymerase subunit β′	LIC10754/*rpoC* gi|447179210	158.0	2	2	87
DNA polymerase III β subunit	LIC10002/*dnaN* gi|24753762	41.5	6	2	91
Glyceraldehyde-3-phosphate dehydrogenase	LIC12090/*gapA* gi|446118864	36.8	14	3	228
Dihydrolipoamide dehydrogenase	LIC11159 gi|447194410	50.4	4	2	103
Glutamine synthetase	LIC12407/*glnA* gi|446013910	53.4	23	20	1333
Acyl-ACP-UDP-N-acetylglucosamine O-acyltransferase	LIC13154/*lpxA* gi|446613534	28.3	56	50	2693
Proton-translocating transhydrogenase subunit α part 1	LIC10046/*pntA* gi|45599193	41.4	12	2	81
ABC transporter ATP-binding protein	LIC11858 gi|446355260	28.0	4	2	45
CusA/CzcA family heavy metal efflux RND transporter	LIC12224 gi|4565873	120.7	2	2	159
Conserved hypothetical protein (formylglycine-generating sulfatase enzyme)	LIC10984 gi|446485100	37.4	13	11	666
Hypothetical protein	LIC11848 gi|45659245	32.1	8	2	47
Hypothetical protein	LIC13428 gi|45659245	54.9	7	3	59

[a]Gene ID was based on ORFs of the genome sequence of *L. interrogans* serovar Copenhageni deposited in GenBank under accession numbers AE016823 (chromosome I) and AE016824 (chromosome II) [39]. [b]Theoretical molecular mass (kDa) was determined by Mascot. [c]Represent MS/MS ion scores determined by peptide mass fingerprinting. Only scores that were deemed to be significant by Mascot analysis ($p < 0.05$) are given.
